# Editorial: Tracing loneliness in aging: understanding the interplay and exploring innovative interventions

**DOI:** 10.3389/fpsyt.2026.1904840

**Published:** 2026-07-30

**Authors:** Madia Lozupone, Mario Altamura, Antonello Bellomo, Francesco Panza

**Affiliations:** 1 Department of Translational Biomedicine and Neuroscience “DiBraiN”, University of Bari Aldo Moro, Bari, Italy; 2 Psychiatric Unit, Department of Clinical and Experimental Medicine, University of Foggia, Foggia, Italy; 3 Dipartimento Interdisciplinare di Medicina, Clinica Medica e Geriatria “Cesare Frugoni”, University of Bari Aldo Moro, Bari, Italy

**Keywords:** aging, depression, digital intervention, frailty, loneliness, social isolation

## 1 Conceptual framework of loneliness/social isolation

Population aging is one of the most important demographic transformations of the twenty-first century. Alongside increased longevity, older adults face growing challenges related to social participation, mental health, cognitive functioning, and quality of life. Loneliness and social isolation have emerged as major public health concerns because of their associations with frailty, depression, cognitive decline, disability, and all-cause mortality (1). Although loneliness reflected the subjective perception of insufficient social relationships and social isolation referred to objective deficits in social connections (2), both frequently coexisted and interacted within the broader biopsychosocial framework of aging (3).

The present Research Topic aimed to deepen understanding of the mechanisms linking loneliness, social isolation, frailty, cognitive decline, and mental health in older age. In particular, it sought to explore how biological, psychological, social, and technological determinants may interact to shape vulnerability and resilience among older adults. Contributions addressing assessment strategies, intervention models, longitudinal pathways, and preventive approaches were especially relevant within this framework.

## 2 Psychosocial and functional determinants

**Figure 1:**
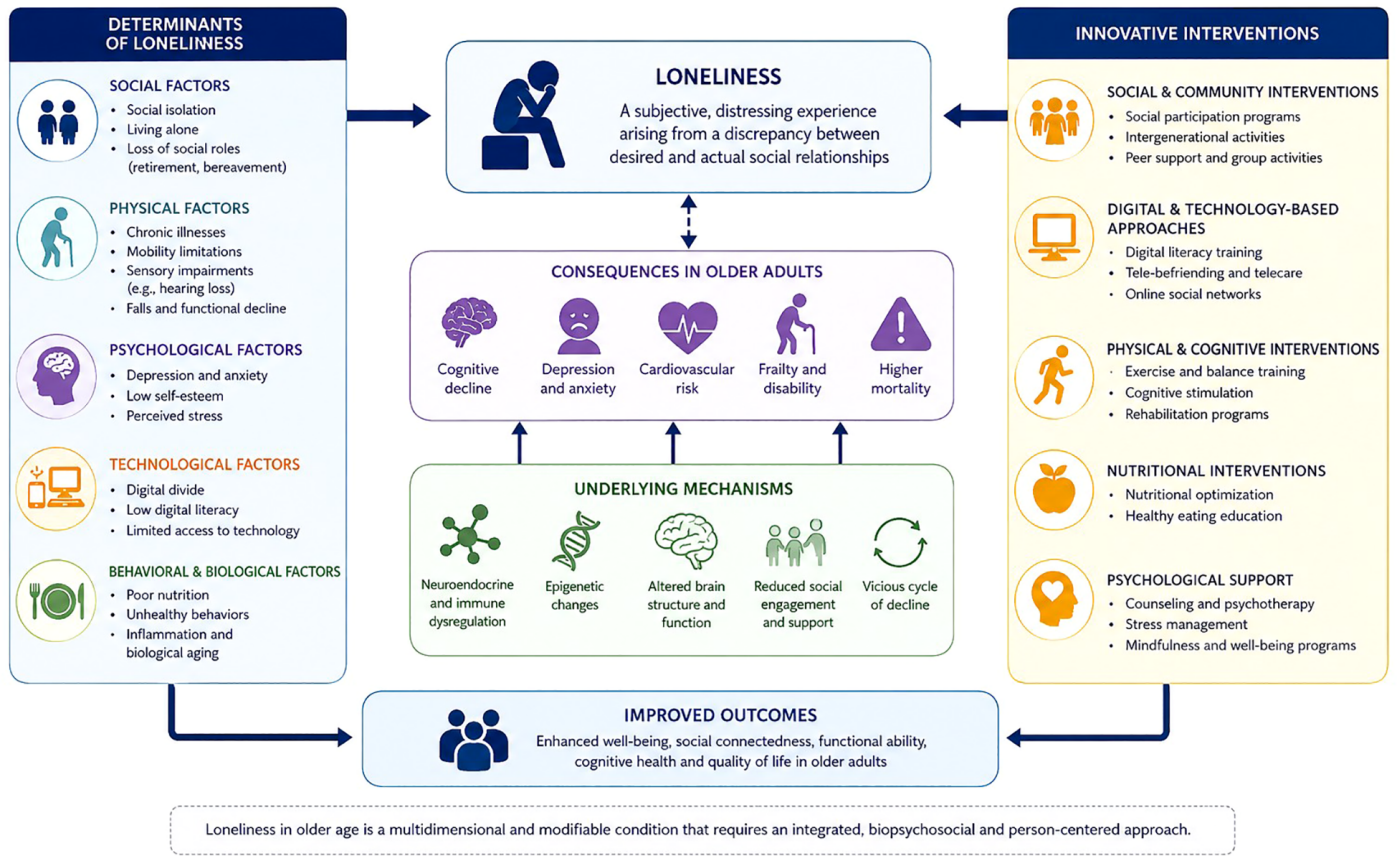
Loneliness in aging: interplay, consequences, and innovative interventions.

Several studies included in the present Research Topic illustrated the multifactorial determinants of loneliness and social isolation (**Figure 1**). Falls were associated with increased loneliness through impaired social adaptation (Yu et al.), bidirectional emotional support reduced social isolation partly through lower depressive symptoms (Yang et al.), and retirement-related loss of social roles contributed to depression through deteriorating physical health and interpersonal relationships (Zhang et al.). Collectively, these findings highlighted the importance of functional ability, emotional connectedness, and meaningful social participation in promoting resilience during aging.

## 3 Frailty, cognition, and biological vulnerability

The role of functional decline and frailty in shaping loneliness trajectories is increasingly recognized. Chronic illnesses, mobility limitations, sensory impairments, and reduced autonomy may progressively restrict opportunities for social participation and community engagement (4). In turn, loneliness may further contribute to unhealthy behaviors, reduced physical activity, and accelerated disability, creating a vicious cycle between psychosocial vulnerability and physical decline (5). Integrating loneliness within a biopsychosocial frailty framework therefore allows for a more comprehensive understanding of the reciprocal pathways linking physical health, cognitive functioning, emotional well-being, and social participation.

A second group of studies within the present Research Topic highlighted the close interaction between physical health, cognition, and social connectedness. Persistent demoralization among older adults with end-stage kidney disease was associated with poor social support and reduced functional independence (Yu et al.) Social isolation and poor nutritional status showed synergistic associations with cognitive decline (Li et al.), while hearing impairment emerged as an important contributor to both cognitive vulnerability and social isolation (Dhanda et al.) Complementing these findings, (Skrzynski and Bryan) demonstrated that older adults who engaged in solitary alcohol consumption experienced poorer subjective and objective cognitive functioning than those drinking primarily in social contexts, emphasizing the importance of social engagement for cognitive health. Additional evidence demonstrated associations between loneliness and hypertension (Xiao et al.,) whereas higher levels of social interaction predicted better functional status during the COVID-19 pandemic (Zhang et al.) Severe self-neglect also clustered with depression, loneliness, and reduced social support (Yi et al.) Together, these findings reinforced the concept of multidimensional models of frailty in which physical, cognitive, emotional, and social factors were closely interconnected (6, 7), with important implications for risk management of neuropsychiatric syndromes in older age.

Beyond the quantity of social interactions, their quality also matters. Szameitat et al. demonstrated that aging may alter recognition of social information conveyed through laughter, suggesting that age-related changes in social cognition could contribute to interpersonal difficulties and vulnerability to loneliness. Complementing this perspective, the COMPASS-HOA study protocol by Tang et al. proposed a multilevel intervention combining psychological support, interpersonal relationship enhancement, community engagement, volunteer involvement, and technology-assisted communication for lonely older adults living in poverty. Together, these contributions emphasized that loneliness is a multidimensional phenomenon requiring both social-cognitive understanding and coordinated multilevel interventions.

Within this perspective, assessment strategies for loneliness in older adults may represent a major challenge. Although several validated scales exist, the subjective and multidimensional nature of loneliness complicates its evaluation in both clinical and research settings. Accurate assessment requires consideration not only of emotional and social dimensions but also of functional status, cognitive impairment, depressive symptomatology, and cultural context. Furthermore, longitudinal approaches are essential for understanding how loneliness evolves across different stages of aging and how it interacts with multimorbidity, disability, and frailty over time.

## 4 Digital inclusion and technology

Another important dimension addressed in the present Research Topic concerned the growing role of technology-mediated interactions and digital health interventions. The rapid digitalization of social and healthcare systems has created both opportunities and challenges for older populations (8). On the one hand, digital technologies and telemedicine may facilitate communication, healthcare access, and social participation, especially for individuals with mobility limitations or living in geographically isolated areas. On the other hand, older adults often face barriers related to digital literacy, socioeconomic inequalities, cognitive decline, and limited access to technological resources.

The growing role of digital technologies emerged as a recurring theme across several contributions of the present Research Topic. Internet use was associated with higher well-being, lower depression and loneliness, greater social engagement, and reduced affective disorders among older adults in different cultural settings. Social participation appeared to mediate many of these benefits, suggesting that digital connectivity may promote healthy aging primarily by strengthening social relationships. At the same time, digital inequalities, limited digital literacy, and increased exposure to digital risks remain important challenges (Dahl Aslan et al.) Findings from studies conducted in China (Kuang et al.; Lu et al.; Miao et al.), Ireland (Jiao et al.), the Czech Republic and Slovenia (Klun et al.), collectively supported the integration of digital inclusion strategies within broader healthy aging policies.

## 5 Interventions and future directions

Intervention research increasingly supported multidomain approaches (**Figure 1**). Programs combining physical activity, cognitive stimulation, nutritional education, social participation, digital literacy, and intergenerational exchange demonstrated promising effects on emotional well-being, social connectedness, and healthy aging (Cossu et al.) Similarly, tele-befriending and community-based initiatives suggested that scalable hybrid models integrating in-person and technology-mediated support may help address loneliness in diverse older populations (Lim et al.; Jönsson et al.).

These findings were especially relevant in light of broader societal transformations affecting older adults worldwide. Urbanization, changing family structures, migration patterns, and the increasing reliance on digital communication have profoundly altered traditional forms of social interaction and caregiving. At the same time, the COVID-19 pandemic highlighted both the vulnerability of older adults to social disconnection and the potential role of technology in maintaining social engagement during periods of crisis and physical distancing. Together, these studies emphasized that maintaining social participation—whether through sensory health support, digital inclusion, or opportunities for meaningful interpersonal interaction—may represent a critical strategy for promoting psychological resilience and healthy aging. Hybrid care models combining in-person interactions with virtual support may therefore represent an important direction for future public health strategies. Emerging evidence also suggested that loneliness may influence biological pathways associated with aging and mental health (9). Chronic loneliness has been associated with inflammatory dysregulation, neuroendocrine stress responses, immune dysfunction, and epigenetic mechanisms potentially implicated in late-life depression and cognitive decline (9).

These biological processes further supported the inclusion of loneliness as a multidimensional domain within frailty models and emphasized the importance of early prevention and intervention (10). Addressing loneliness in older age required coordinated and interdisciplinary efforts involving clinicians, psychologists, gerontologists, public health professionals, social workers, policymakers, caregivers, and technology developers. Effective interventions should move beyond simply increasing social contact and instead foster meaningful, reciprocal, and emotionally supportive relationships while also promoting autonomy, social participation, physical functioning, and digital inclusion. Ultimately, loneliness should no longer be regarded as an inevitable consequence of aging but rather as a modifiable biopsychosocial condition demanding comprehensive scientific, clinical, and societal attention. By integrating evidence from diverse methodological and disciplinary perspectives, the present Research Topic aimed to advance understanding of the complex relationships among loneliness, frailty, cognitive decline, emotional well-being, and social participation in later life.

We hope that the contributions included in this collection will stimulate interdisciplinary dialogue, inform public health policies, and support the development of innovative interventions capable of promoting healthy aging, resilience, and social connectedness among older adults worldwide. Collectively, the studies included in the present Research Topic demonstrated that loneliness and social isolation were not isolated psychosocial experiences but complex multidimensional phenomena affecting physical health, cognitive functioning, emotional well-being, and social participation. Evidence highlighted the importance of adopting integrated biopsychosocial approaches combining assessment, prevention, digital inclusion, social engagement, and multilevel interventions. As populations continue to age, addressing loneliness and social isolation should remain a central priority for clinical practice, public health policy, and future research aimed at promoting resilience, well-being, and healthy aging.
